# Lightweight and hybrid transformer-based solution for quick and reliable deepfake detection

**DOI:** 10.3389/fdata.2025.1521653

**Published:** 2025-04-01

**Authors:** Geeta Rani, Atharv Kothekar, Shawn George Philip, Vijaypal Singh Dhaka, Ester Zumpano, Eugenio Vocaturo

**Affiliations:** ^1^Manipal University Jaipur, Jaipur, Rajasthan, India; ^2^Department of Computer Engineering, Modeling, Electronics and Systems (DIMES), University of Calabria, Rende, CS, Italy; ^3^National Research Council, Institute of Nanotechnology (NANOTEC), Rende, CS, Italy

**Keywords:** deepfake, social safety, transformer, blackmail, computation, generative

## Abstract

**Introduction:**

Rapid advancements in artificial intelligence and generative artificial intelligence have enabled the creation of fake images and videos that appear highly realistic. According to a report published in 2022, approximately 71% of people rely on fake videos and become victims of blackmail. Moreover, these fake videos and images are used to tarnish the reputation of popular public figures. This has increased the demand for deepfake detection techniques. The accuracy of the techniques proposed in the literature so far varies with changes in fake content generation techniques. Additionally, these techniques are computationally intensive. The techniques discussed in the literature are based on convolutional neural networks, Linformer models, or transformer models for deepfake detection, each with its advantages and disadvantages.

**Methods:**

In this manuscript, a hybrid architecture combining transformer and Linformer models is proposed for deepfake detection. This architecture converts an image into patches and performs position encoding to retain spatial relationships between patches. Its encoder captures the contextual information from the input patches, and Gaussian Error Linear Unit resolves the vanishing gradient problem.

**Results:**

The Linformer component reduces the size of the attention matrix. Thus, it reduces the execution time to half without compromising accuracy. Moreover, it utilizes the unique features of transformer and Linformer models to enhance the robustness and generalization of deepfake detection techniques. The low computational requirement and high accuracy of 98.9% increase the real-time applicability of the model, preventing blackmail and other losses to the public.

**Discussion:**

The proposed hybrid model utilizes the strength of the transformer model in capturing complex patterns in data. It uses the self-attention potential of the Linformer model and reduces the computation time without compromising the accuracy. Moreover, the models were implemented on patch sizes of 6 and 11. It is evident from the obtained results that increasing the patch size improves the performance of the model. This allows the model to capture fine-grained features and learn more effectively from the same set of videos. The larger patch size also enables the model to better preserve spatial details, which contributes to improved feature extraction.

## 1 Introduction

The rise of Artificial Intelligence (AI) based image generative techniques such as Generative Adversarial Networks (GANs), diffusion models, neural style transfer, and free swap applications is a major cause of generating fake images and videos of people. The ease of accessibility of such applications is the primary reason for the boom in the number of fraudulent cases using deepfake technology. Floating such videos on social media platforms are causes of concern. A report shows that the number of deepfake videos available online is growing exponentially (Tyagi and Yadav, 2023). Such videos and images may be used to blackmail, or fraud. In addition, pornographic content was generated from various public figures using image generation models to tarnish their reputation (Westerlund, 2019). Manipulation of common people using deepfakes of their close relatives is another issue observed in many countries (Zandt, 2024). The report published in 2022 (Doss et al., 2023), shows that 71% of people are unaware of the deepfake and rely on such videos. Therefore, there is an urgent need for deepfake detection. Researchers proposed various techniques for deepfake detection. These techniques are based on analyzing facial and body movements to indicate manipulation, lighting changes, or inconsistencies in images, expressions, or audio-visual cues. For example, Lin et al. (2023) addressed the problem of deepfake detection even on low-quality image datasets. They proposed a multi-scale convolution based on EfficientNet and the vision transformer model for deepfake detection. Their multi-scale module can effectively capture the face details at different scales and enhance the detection performance of the proposed model. They employed their model on Celeb DF-v2 dataset (Li et al., 2020) to prove their efficacy in deepfake detection. These models may stuck in local optima and may report lower accuracy. The researchers in Ewees et al. (2024) proposed a feature selection method to minimize the problem of local optima. Another research group, Ghorbanpour et al. (2023) proposed a transformer-based detection algorithm for fake news detection using text and images. Next, Ramadhani et al. employed Video Vision Transformer (ViViT) architecture for video deepfake detection. The system reported 92.52% F1 score (Ramadhani et al., 2024) on the Celeb-DF version 2 dataset (Li et al., 2020). ViT models can capture global dependencies in images, allowing them to understand relationships between distant image regions. Also, their computational cost is primarily dependent on the number of patches rather than the entire image. The ViT models can be computationally expensive for large-scale datasets and high-resolution images. Linformer, a variant of the transformer architecture, is designed to address the quadratic computational complexity of self-attention mechanisms in transformers. However, it may result in a loss of expressive power, potentially affecting the model's ability to capture complex patterns in data. The effectiveness of Linformer may vary depending on the specific task and dataset, requiring careful tuning and experimentation.

It is evident from the above discussion that existing techniques lack generalization and robustness. So, these are not efficient in detecting deepfake content generated by different techniques. Also, most of the techniques are proposed to detect deepfake images, audio, and text. This leaves a scope for deepfake video detection. The techniques proposed so far use Convolutional Neural Networks (CNNs), or transformer, or Linformer models for deepfake detection. As mentioned above, these techniques have advantages and challenges. Thus, there is scope to introduce techniques that are computationally less expensive and accurate in deepfake detection. In this paper, the above-identified gaps are minimized, and a hybrid model for deepfake video detection is introduced. The model is an integration of the Vision transformer (ViT) and Linformer models. The proposed model is fine-tuned to reduce its computation cost. The major contributions of this manuscript are as follows:

- To develop a hybrid model by integrating transformer and Linformer models for video deepfake detection.
- To improve robustness and generalization ability of deepfake detection techniques.
- To reduce the computation cost of deepfake detection without compromising accuracy.

Following the introduction, the related works examine previous studies relevant to deepfake detection. The methodology section outlines the preparation of the data, the architecture of the proposed model, the training of the model, and the evaluation metrics used to evaluate the performance of the model. Subsequently, the results section presents the findings derived by testing the model. Finally, the conclusion section summarizes the contributions to the field and suggests practical applications.

## 2 Related works

In this section, we discuss the available literature in the domain of deepfake detection. We elaborate on contributions of various researchers and highlight the limitations that need to be addressed. Enes et al. presented a review of deepfake techniques including definitions, datasets, performance metrics, and standards employed in existing works (Altuncu et al., 2024). Heo et al. integrated CNN features with patch-based positioning within a vision transformer framework. This integration leverages the strengths of both CNNs and transformers, specifically to capture local artifacts more effectively. Additionally, they introduced a distillation process where a distillation token, trained using binary cross-entropy through a sigmoid function, helps in improving the generalization of the model. This hybrid approach reported an accuracy of 82.63%. However, it is effective in deepfake detection, addressing both local and global feature extraction, there is a scope for improving the accuracy of detection (Heo et al., 2023). Also, the model is applied for fake image detection, leaving the scope to work on fake video detection.

Usman et al. proposed a novel approach for detecting deepfake images using a shallow vision transformer model. They divided the images into non-overlapping patches, and utilized a softmax layer for classification. Their model reported the AUC value of 95.9% and F1 score of 91.9% in distinguishing between authentic and manipulated images on the DFDC dataset (Dolhansky et al., 2020). Their approach proves the efficiency of the shallow vision transformer, particularly in scenarios with limited training data and computational resources. There is further scope to improve generalization ability of the model so that it can work on images or videos generated by various techniques. Also, the work can be enhanced by training it on geometric and appearance features to differentiate real and manipulated images (Usmani et al., 2024).

Ghorbanpour et al. introduced the Fake News Revealer (FNR) algorithm for the detection of fake news in social media. The algorithm incorporates a ViT model to extract semantic and contextual features from images in news content. Their approach utilizes a multi-modal approach to detect fake news by integrating textual and visual information. Experimental results reported on publicly available social media news datasets comprising tweets demonstrate the superior performance of the FNR algorithm (Ghorbanpour et al., 2023).

Lin et al. proposed a new CNN-based method for deepfake detection. The method combines a multi-scale module and an MBConv block module in a dual-subnet structure. The multi-scale module captures face details using dilation convolutions, while the MBConv block module learns high-dimensional face semantic information. The proposed method also introduced a vision transformer module for final classification. The authors carefully analyzed and designed each module of the proposed model to enhance the detection performance (Lin et al., 2023). Next, Coccomini et al. proposed the use of mixed convolutional-transformer networks for video deepfake detection. They combined pre-trained convolutional networks, such as EfficientNet B0, with ViT model. The model achieved F1 score of 88.0%, which is close to the state-of-the-art methods on the DFDC (Dolhansky et al., 2020) and FaceForensics++ datasets (Rössler et al., 2019). They did not use distillation or ensemble techniques to keep the model simple. The authors also introduced a voting scheme for inferring the presence of deepfakes in videos with multiple faces (Coccomini et al., 2022). This technique utilizes all possible landmarks for deepfake detection, so is computationally intensive.

Ramadhani et al. presented a research on video deepfake detection based on Video Vision Transformer (ViViT) architecture. The system utilizes landmark area images as input and extracts spatiotemporal features using a combination of Depthwise Separable Convolution (DSC) block and Convolution Block Attention Module (CBAM) from tubelet. Computer Vision and Machine Learning (CVML) in reference to Celeb-DF (v2) dataset signifies that computer can see and understand images and videos. Further the machine learning techniques can be employed to analyze and process the information captured from these images and videos. It achieved an accuracy of 87.18% and an F1 score of 92.52% (Ramadhani et al., 2024).

Passos et al. (2024) presented a review of the latest techniques adopted for deepfake detection. They claimed that robust unsupervised or semi-supervised approaches for deepfake detection need to deal with continuously evolving deepfake contents on social networks. Also, there is a strong demand to minimize the time-consuming manual annotation of data. Further, the accuracy of existing techniques is dependent on deepfake generating methods. The models are ineffective if it is trained on the deepfake dataset generated by one method and tested for another. Thus, there is a need to develop robust models that can recognize deepfake content generated by multiple techniques. Moreover, there are limited studies on deepfake detection from video datasets. Also, the existing techniques are computationally intensive.

Thus, it is apparent from the above discussion that deepfake videos and images can be generated by multiple variants of autoencoders, and generative adversarial networks (GANs). These techniques can generate deepfake videos nearly indifferentiable from real ones. Most of the methods proposed in the literature focus on deepfake image detection. Thus, there is a need to address the issue of deepfake video detection. Moreover, the existing techniques are computationally intensive which raises the need for models having low training time and high accuracy of deepfake detection. To address these challenges, the researchers in this manuscript proposed a hybrid model comprising the vision transformer and the linformer models. To improve generalizability and robustness, they trained the model on the dataset comprising videos collected from various sources with differences in contrast, background, and lighting etc.

## 3 Methodology

This section elaborates on the dataset used, the architecture of the proposed model, training, and testing mechanisms of the model.

### 3.1 Dataset

The Celeb DF-v2 dataset (Li et al., 2020) has been used to train both the ViT and hybrid model developed by integrating the ViT and Linformer models. The dataset consists of 590 real videos and 5,639 deepfake videos. For training the ViT and hybrid models, a dataset comprising 1,000 images randomly selected from The Celeb DF-v2 dataset (Li et al., 2020) was prepared. This dataset is balanced and contains an equal number of real and fake videos. The real videos feature a diverse set of celebrities, ensuring a wide range of facial attributes, expressions, and lighting conditions. Each video varies in duration from a few seconds to several minutes. These videos include various backgrounds and motions, providing a realistic and challenging dataset for deepfake detection. The videos are divided into frames for deepfake detection.

### 3.2 Architecture of proposed model

The deepfake detection system proposed in this research utilizes the Vision Transformer (ViT) architecture as a base model (Al-hammuri et al., 2023). The model consists of the pre-processing phase and the detection phase. To reduce the execution time and improve accuracy, a Linformer (Linear Transformer) (Wang et al., 2020), an efficient variant of standard transformers is integrated. The details of the architectures are given in the following subsections.

#### 3.2.1 Vision transformer

This model is designed specifically for computer vision tasks. An input image given to the model is represented with height (H), width (W), and number of channels (C). The attention mechanism used in ViT is defined in Equation 1.


(1) $$\begin{array}{l} Attention\left(Q,K,V\right)=softmax\left(\frac{QK^{T}}{\sqrt{D}}\right)V \end{array}$$

In Equation 1, Q, K, V, and D are queries, keys, values, and dimensions of the input embeddings respectively.

**Figure 1:**
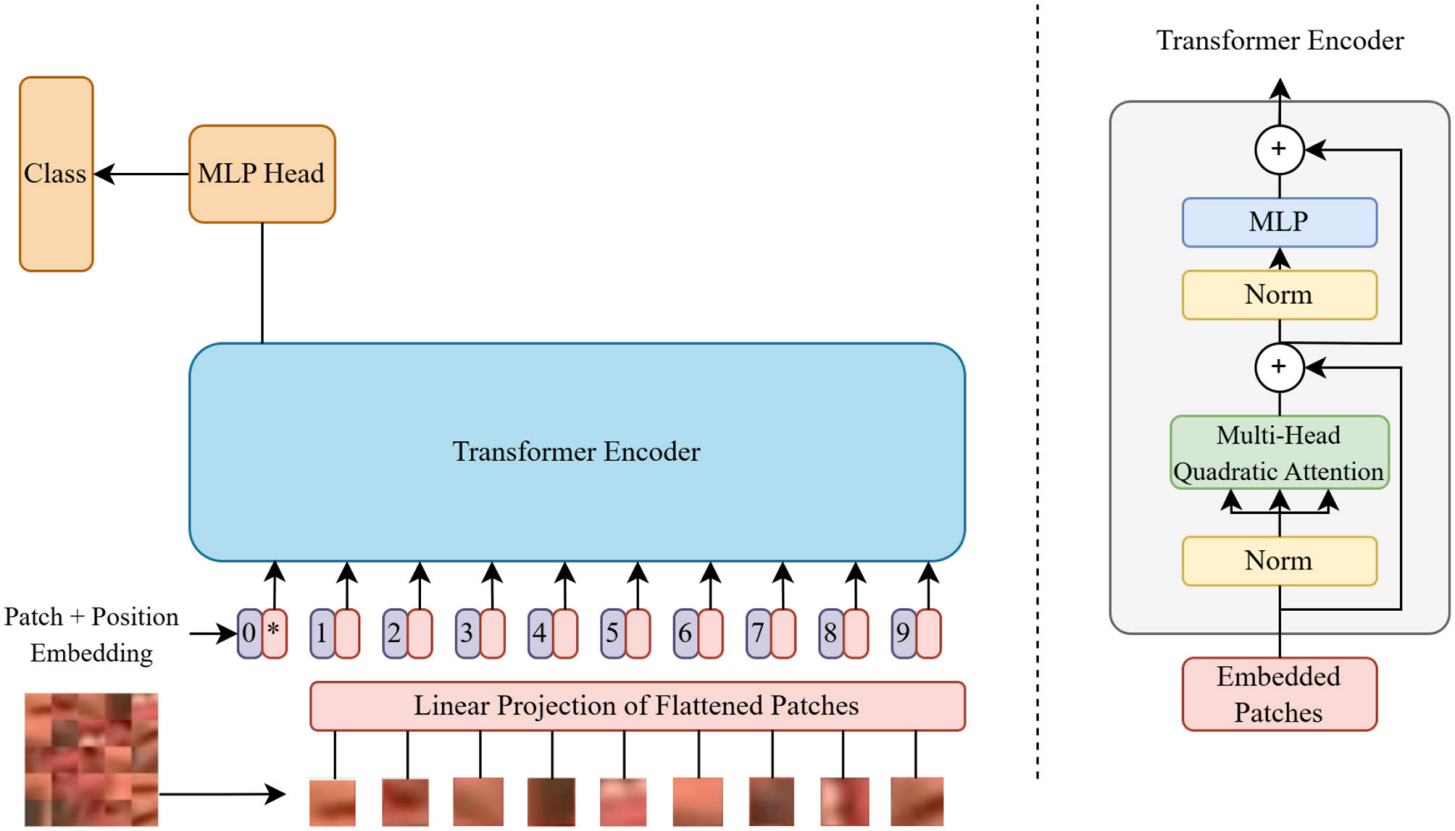
Architecture of vision transformer model.

Initially, the input image is converted into patches using a rearrangement operation, followed by layer normalization. Next, a linear projection to the transformer dimension is applied to the projected embeddings, accompanied by another layer normalization. Positional information is then added to the patch embeddings via a positional embedding layer, and a class token is introduced for classification tasks. The sequence of patches and the class token are processed by the transformer module. After this, mean pooling is applied to the transformer's output. Finally, the model's output is normalized and projected to class scores using a multilayer perceptron (MLP) head, consisting of a layer normalization and a linear layer. The architecture of the ViT model is shown in Figure 1, and its various components with their functionalities are explained below.

- Linear Projection of Flattened Patches: The representation of patches as vectors is flattened and then linearly projected to obtain embeddings with a higher-dimensional representation. It helps the model learn complex relationships between different parts of the input.
- Position Embeddings: These are a set of vectors for each patch location that is trained with gradient descent along with other parameters. These are added to the patch embeddings because the patches by themselves do not have any information about their exact origin.
- Transformer Encoder: It consists of multiple layers of self-attention mechanisms followed by feed-forward neural networks. This is responsible for capturing contextual information from the input patches.
- Multi-Head Attention: Instead of performing a single operation, multiple operations are run in parallel. Each head has its own set of Q, K, and V projections as shown in Equation 1, allowing the model to capture different aspects of relationships between the patches.
- Multi-Layer Perceptron (MLP) Head: It is a simple feed-forward neural network consisting of several dense layers. It includes a non-linear activation function. It employs the Gaussian Error Linear Unit (GeLU) activation function instead of the Rectified Linear Unit (ReLU) activation function. This function can adapt more effectively to different input scales. It ignores neurons with negative values, so diminishes the vanishing gradient problem as encountered in ReLU.

#### 3.2.2 Linformer

**Figure 2:**
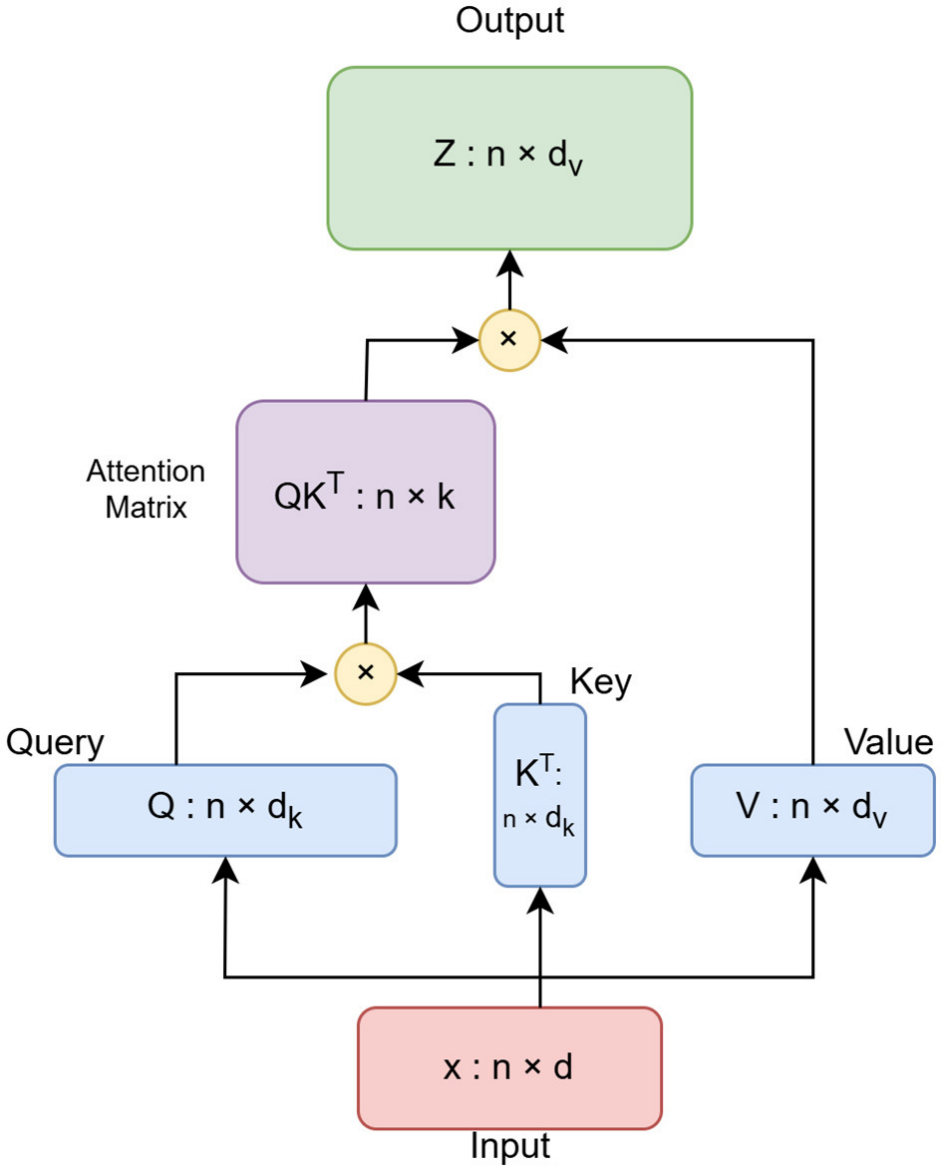
Architecture of Linformer model.

Linformer is the Self-Attention with Linear Complexity. The transformer module used in the ViT model is specified as an instance of the Linformer class, an efficient variant of transformers (Wang et al., 2020). In this study, the sequence length is changed from 64 to 65. The sequence length denotes the total number of patches and one class token. The extra class token is required for the Linformer architecture. The standard attention self-mechanism of the transformer incurs O(n^2^) time complexity. Its space and time complexity are directly dependent upon the sequence length. Linformer approximates this self-attention by a low-rank matrix. Thus, it reduces the execution time to half without compromising the accuracy. The architecture of the Linformer model is shown in Figure 2. The attention scores are also computed using a reduced-dimensional key matrix as defined in Equation 2.


(2) $$A^{\prime }=\left(\frac{Q{K^{\prime }}^{T}}{\sqrt{D}}\right)V$$

In Equation 2, the original key matrix K is projected to a lower dimension to reduce the size of the attention matrix. K', and Q are the projected key matrix, and query matrix respectively. D is the dimensionality of the queries and keys, and A' is the resulting attention matrix after normalization. Thus, the complete attention mechanism in Linformer is represented in Equation 3.


(3) $$Attention(Q,K^{\prime },V^{\prime })=softmax\left(\frac{Q{K^{\prime }}^{T}}{\sqrt{D}}\right)V^{\prime }$$

#### 3.2.3 Hybrid transformer model

**Figure 3:**
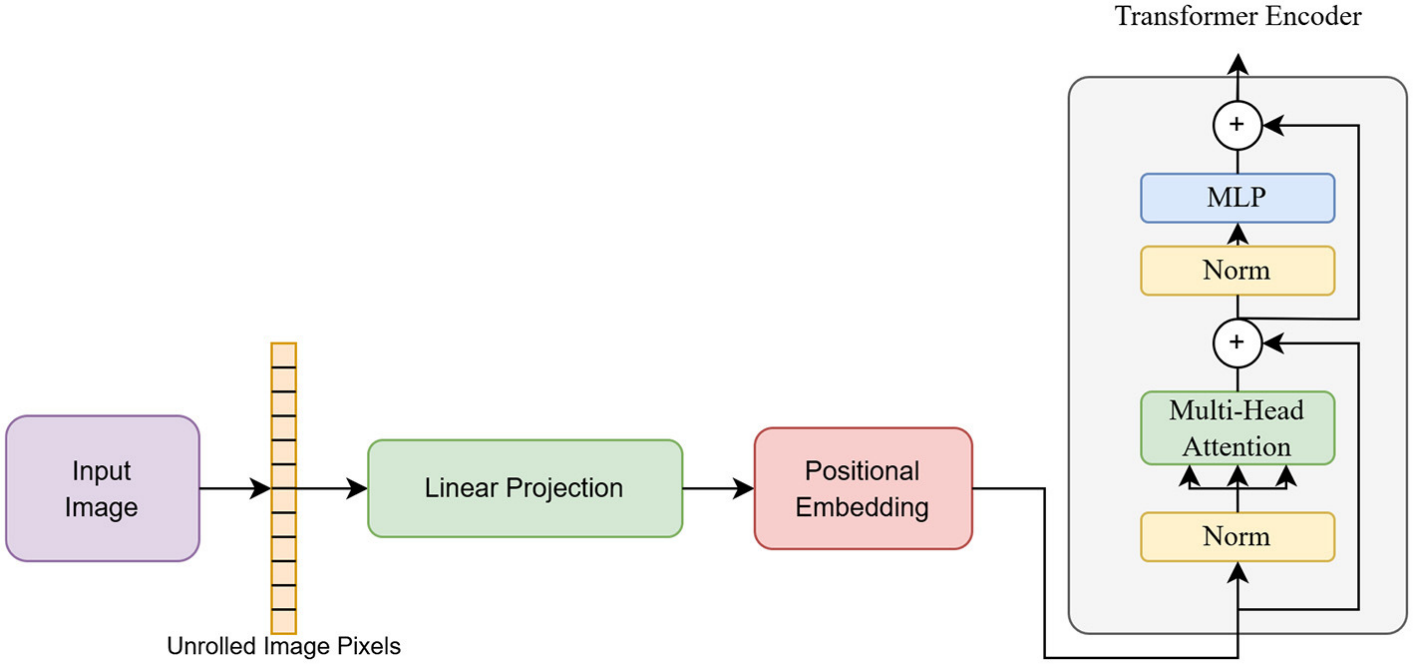
Architecture of hybrid transformer model.

The ViT and Linformer models have been integrated to reduce computational overhead while preserving strong feature extraction capabilities. In the proposed architecture, standard multi-head self-attention (MHSA) is replaced with Linformer's efficient self-attention. Here, the key (K) and value (V) matrices are projected using a low-rank decomposition, reducing dimensions while retaining essential information. This transformation reduces ViT's quadratic scaling to linear complexity, thereby decreasing memory usage. The block diagram of the hybrid architecture is presented in Figure 3. As shown in the figure, the input image is divided into patches, which are then linearly projected and encoded with positional information. The encoded patches are subsequently passed to the encoder of ViT for classification into fake and real images.

### 3.3 Training

**Figure 4:**
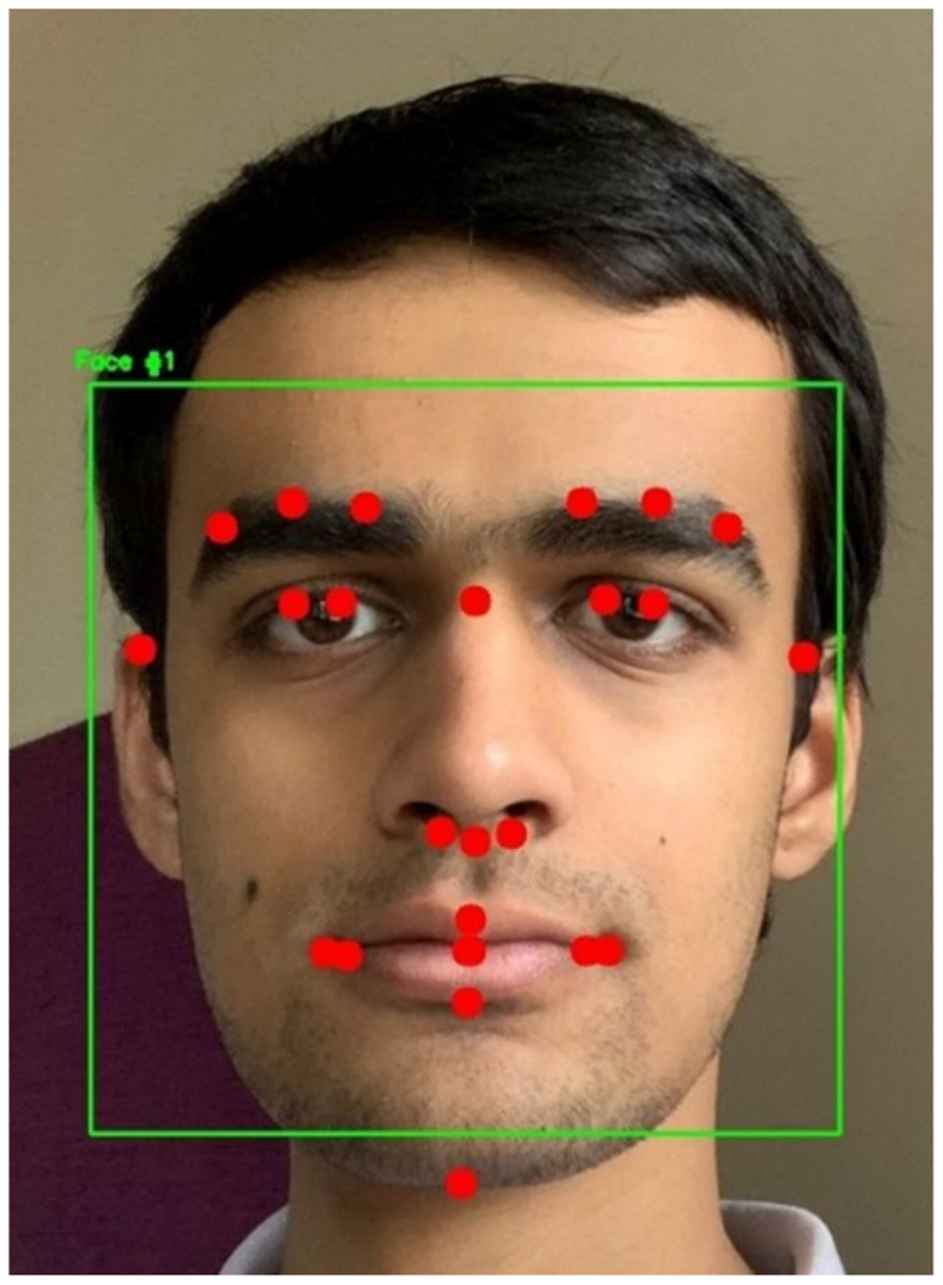
Sample frame with drawn bounding box and facial landmarks.

The ViT and the proposed hybrid model of ViT and Linformer models were implemented using Python 3.12 and PyTorch 2.0. The code was executed on a machine with 64 GB RAM, Intel^®^ Core™ i9-10900K CPU @ 3.70 GHz, GeForce RTX 3080 Ti, and a disk space of 3 TB. The system used the Ubuntu 22.04 operating system. Initially, the dlib library (Davis, 2009) which includes models pre-trained on the iBUG 300-W dataset (Sagonas et al., 2016), was used for facial landmark detection. The dataset consists of 7,764 images and was originally designed to detect 68 facial landmarks. However, due to its high computational complexity, a custom shape predictor (Yang et al., 2019) was developed to identify 25 essential landmarks specifically for facial recognition, and reducing computation time. For this study, 1,000 randomly selected videos from the Celeb-DF v2 dataset were analyzed. The highest AUC scores were reported when landmarks were selected from the central region of the face. This is due to inconsistencies introduced by variations in head poses. The dataset was manually annotated with 68 landmark coordinates, stored as (x, y) integer pairs. An XML file contained the bounding box coordinates of faces in the images, allowing access to specific facial features such as the mouth, nose, eyes, eyebrows, and jaw using predefined XML elements. The custom selection of 25 landmarks was used to create the training and testing datasets. The custom shape predictor model produced a 36 MB .dat file. A sample frame, with a bounding box around the detected landmarks, is shown in Figure 4. Dlib also employs the Histogram of Oriented Gradients (HOG) algorithm along with a Support Vector Machine (SVM) for classification. However, it fails to detect faces in all frames. To address this limitation, a CNN-based model was used to detect faces that Dlib could not recognize. In the next phase, the videos from Celeb-DF v2 (Li et al., 2020) were converted into individual frames. Each detected face was marked with 25 facial landmarks, and for each landmark, an 11 × 11-pixel region centered on the landmark was extracted. These 25 small images were then concatenated.

Further, Dlib (Dadi and Pillutla, 2016) uses the Histogram of Gradients (HOG) algorithm and Support Vector Machine (SVM) for classification. But, it fails to detect faces in all the frames. Therefore, a CNN model has been used to detect faces that were not recognized by Dlib.

In the next phase, the videos from the Celeb DF-v2 dataset were divided into individual frames. Landmarking of 25 points was done on the faces in each frame. For each landmark, an 11 × 11-pixel area centered around the landmark was extracted. Thus, for 25 landmarks, an image of 55 × 55 was obtained for each frame after concatenating the 25 resulting images. The concatenation was done to emphasize the features rather than the entire frame. Such marked frames were given as input to the ViT the hybrid of ViT and Linformer models to classify images into fake and real categories. These models are trained with a batch size of 128, a learning rate of 10e-4, and a seed of 142. The models are employed with the Adam optimizer, and cross entropy loss function, and trained for 50 epochs. After 50 epochs, there is no decrease in the value of loss function which indicates the models do not require more training.

### 3.4 Evaluation metrics

The performance of the model has been evaluated in terms of accuracy, loss function, F1 score, and Area under curve (AUC) as defined below. Detailed definitions of these metrics are available in Ankita Gangwar et al. (2024) and Rainio et al. (2024). In addition, the computational complexity of a model was reported in terms of Giga Floating Point Operations (GLOPs).

- Accuracy: As defined in Equation 4, it is the ratio of the number of correct predictions to real and fake classes from the total number of predictions made by the model.

(4) $$\begin{array}{l} Accuracy=\left(\frac{TR+TF}{TR+TF+FR+FF}\right) \end{array}$$

In Equation 4, TR is the number of correct predictions of real images. TF is the number of correct predictions of fake images. FR is the number of incorrect predictions of real images to fake class. FF is the number of incorrect predictions of fake images to real class.

- Loss Function: The value of the loss function represents the error between the actual and predicted results. It also demonstrates the learning behavior of the model during training. The weights of the deep learning model are adjusted during training to find the optimal value of the loss function. In this manuscript, binary cross entropy loss function is used. It is defined in Equation 5. In this equation, *N* is the total number of data points, *t*_*j*_ is the classification value 0 or 1. and *p*_*j*_ is the Softmax probability for the data point.

(5) $$\begin{array}{l} L=-\frac{1}{N}\overset{N}{\underset{j=1}{\sum }}\left[t_{j}\log\left(p_{j}\right)+\left(1-t_{j}\right)\log\left(1-p_{j}\right)\right] \end{array}$$

- F1 score: It evaluates the predictive efficacy of the model based on its class-wise performance rather than overall performance. As defined in Equation 6, it combines the precision and recall scores of a model.

(6) $$F1\;Score=\frac{2*Precision*Recall}{Precision+Recall}$$

- AUC: It measures the two-dimensional area underneath the entire Receiver Operating Characteristic Curve (ROC) from the co-ordinates (0, 0) to (1, 1). It is a graph indicating the performance of a classification model at all thresholds. It plots the TP and FP rates.

## 4 Results

**Figure 5:**
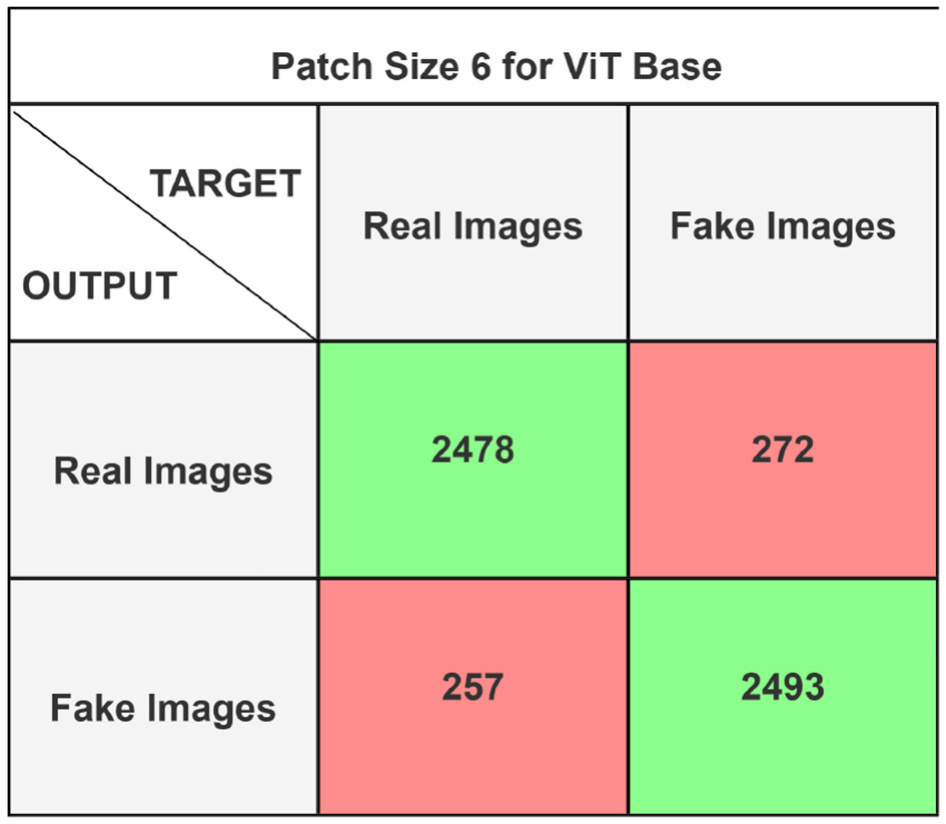
Confusion matrix for ViT base model with patch size 6.

**Figure 6:**
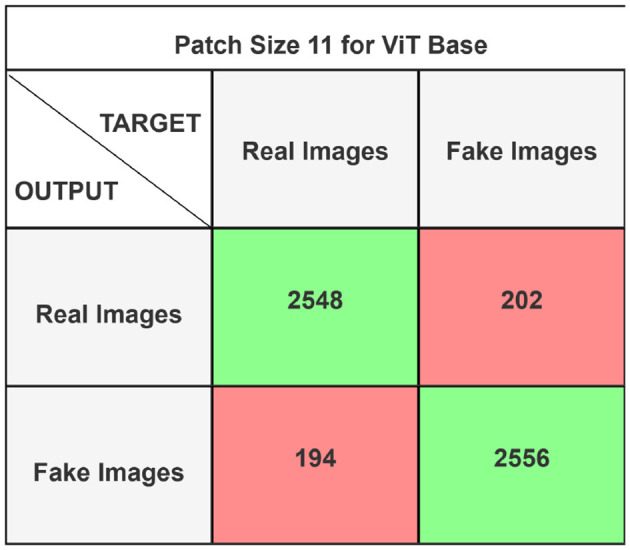
Confusion matrix for ViT base model with patch size 11.

**Figure 7:**
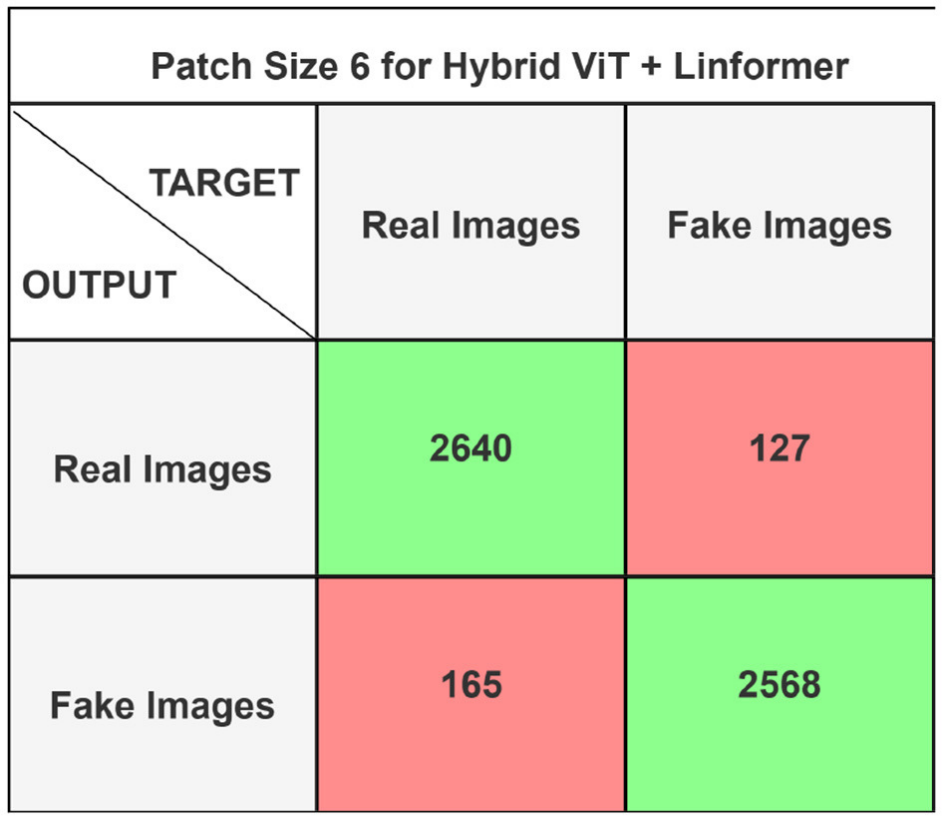
Confusion matrix for hybrid ViT + Linformer model with patch size 6.

**Figure 8:**
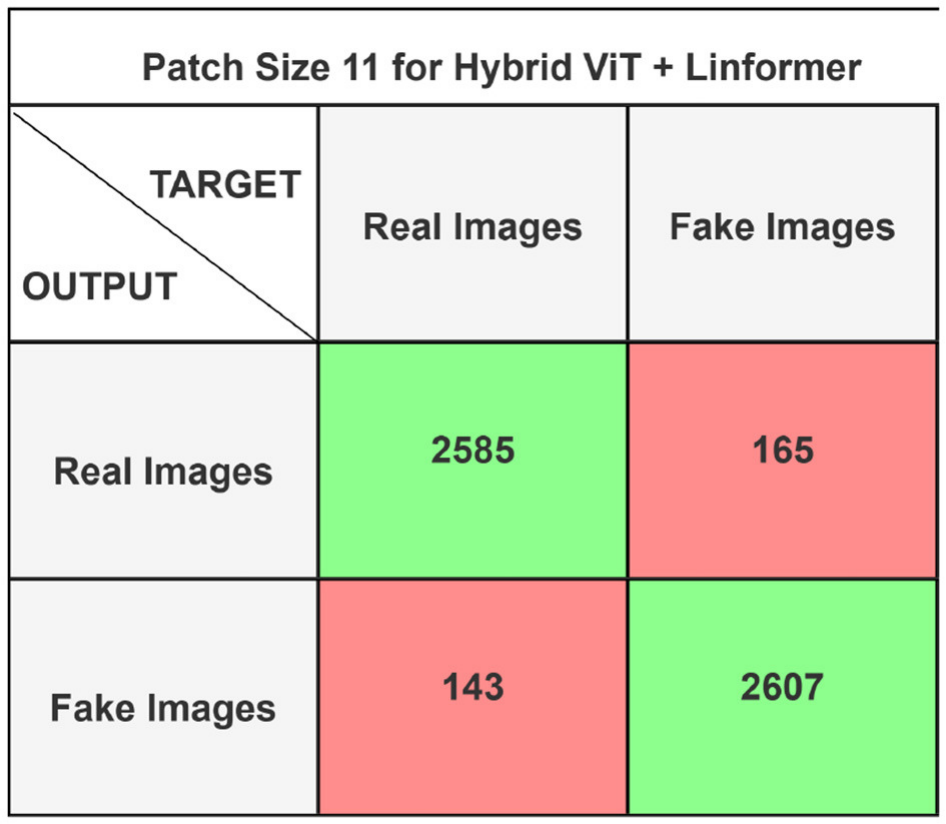
Confusion matrix for hybrid ViT + Linformer model with patch size 11.

The performance of the ViT and the proposed hybrid model have been evaluated on patch sizes of 6 and 11. The patch size was selected arbitrarily to determine its impact on model's performance. From the experimental results shown in Tables 1, 2, it is evident that increasing the patch size improves the performance of the model. A large patch size provides more detailed information from the images. This allows the model to capture fine-grained features and learn more effectively from the same set of videos. The larger patch size also enables the model to better preserve spatial details, which contributes to improved feature extraction. On both patch sizes, the models were executed for 50 epochs. The results were presented using a confusion matrix, accuracy, loss, F1 score, AUC, and Giga Floating Point Operations (GLOPs). Figures 5, 6 illustrate the confusion matrices for the ViT model with patch sizes of 6 and 11, respectively. Similarly, the confusion matrices for the hybrid model are depicted in Figures 7, 8 for the same patch sizes. Additionally, Tables 1, 2 provide the accuracy, loss, F1 score, AUC, and GLOPs for both configurations. It has been observed that the hybrid of ViT and Linformer models converges faster and achieves the maximum accuracy of 98.9% in merely 20 epochs. The results obtained on reducing the patch size by 5 pixels are shown in Table 2. ViT employs a quadratic self-attention mechanism, causing the number of operations to grow exponentially, which increases training time. Additionally, the quadratic computations prolong gradient calculations, slowing down backpropagation. In contrast, a hybrid of ViT and Linformer models accelerates training by leveraging low-rank projections, reducing GLOPs and backpropagation time. Linformer optimizes self-attention through a linear approximation, enhancing memory efficiency and computational speed by replacing quadratic calculations with linear ones. It is evident from the results that reducing the patch size reduces the training time. The training time of the ViT model is reduced by 22.11% when the patch size is decreased from 11 to 6. Similarly, the training time of the proposed hybrid model is reduced by 14.53% when pixel size is reduced from 11 to 6. Moreover, the proposed hybrid model needs 15.32% and 21.4% less time than the vision transformer model when it is trained on pixel sizes 11 and 6 respectively. The reduction in computation time is also supported by the values of GLOPs shown in the last column of Tables 1, 2. The hybrid of ViT and Linformer model reports approximately half values of GLOPs that is 882, and 883 at patch sizes of 6 and 11 respectively. This shows that integrating Linformer model with the ViT model reduces the number of computations. However, there is a negligible change in the accuracy and values of the loss function. The hybrid model achieved a lower loss of 0.1774. This value suggests the hybrid model is better at minimizing errors during training, contributing to its higher accuracy. It is also clear from the results that the hybrid model of ViT, Linformer converges to a better accuracy in lesser epochs than the base ViT model. The hybrid model outperforms the standard ViT by 3.62%. This improvement indicates that the Linformer component helps enhance the model's generalization and prediction capabilities. On a batch size of 6, the hybrid model demonstrated an increase of 4.00% in accuracy, 3.76% in F1 score, and 1.76% in AUC, along with a 24.92% reduction in loss. Similarly, with a batch size of 11, the model showed an improvement of 2.61% in accuracy, 2.03% in F1 score, and 0.41% in AUC. However, it reported a reduction of 17.70% in the loss function value. Thus, it is evident from the above analysis that the hybrid model of ViT and Linformer demonstrates superior performance across all evaluated metrics compared to the ViT model. It achieves higher accuracy, lower loss, and better F1 and AUC scores, all while reducing training time. This proves that the combination of ViT and Linformer leverages the strengths of both architectures, making it a more efficient and effective model for tasks requiring image classification.

**Table 1:** Performance of models using a patch size of 6 pixels.

**Archi-tecture**	**Train time**	**Acc (%)**	**Loss**	**F1**	**AUC**	**GFLOPs (Train)**
Vision trans-former	5h 5m 6s	90.33	0.2363	0.905	0.967	1,962
Hybrid of ViT and Linformer	4h 27m 54s	93.95	0.1774	0.939	0.984	882

**Table 2:** Performance of models using a patch size of 11 pixels.

**Archi-tecture**	**Train time**	**Acc (%)**	**Loss**	**F1**	**AUC**	**GFLOPs (Train)**
Vision trans-former	6h 12m 46s	92.83	0.2751	0.935	0.985	1,963
Hybrid of ViT and Linformer	5h 6m 50s	95.25	0.2211	0.954	0.989	883

## 5 Conclusion

In this manuscript, the objective of correctly detecting deepfake videos generated by various techniques is achieved. The proposed hybrid model utilizes the strength of the transformer model in capturing complex patterns in data. It uses the self-attention potential of the Linformer model and reduces the computation time without compromising the accuracy. The efficacy of the proposed model is also compared with the Vision transformer model used in literature for deepfake detection. The proposed hybrid model reported 21.4% lesser training time than the vision transformer model when both models were trained for 50 epochs on the dataset prepared from the source available at Li et al. (2020). Further, it reported a reduction of approximately 50% in Giga Floating Point Operations (GLOPs). It proves significant improvement in computational efficiency of the proposed model. Moreover, the proposed model achieved robustness and generalizability. It is effective in detecting deepfake videos or images generated by different techniques. The analysis of the loss function values over 50 epochs shows minimal variation, indicating that the model does not exhibit signs of overfitting or underfitting. This stability is a sign of generalization and robustness. Thus, it can be integrated with social media or news platforms etc. to avoid blackmailing or other repercussions in life of people.

## Data Availability

The original contributions presented in the study are included in the article/supplementary material, further inquiries can be directed to the corresponding authors.

## References

[B1] Al-hammuri K. Gebali F. Kanan A. Chelvan I. T. (2023). Vision transformer architecture and applications in digital health: a tutorial and survey. Vis. Comput. Ind. Biomed. Art 6:14. 10.1186/s42492-023-00140-9 37428360 PMC10333157

[B2] Altuncu E. Franqueira V. N. L. Li S. (2024). Deepfake: definitions, performance metrics and standards, datasets, and a meta-review. Front. Big Data. 7:1400024. 10.3389/fdata.2024.1400024 39296632 PMC11408348

[B3] Coccomini D. A. Messina N. Gennaro C. Falchi F. (2022). “Combining efficientnet and vision transformers for video deepfake detection,” in Image Analysis and Processing - *ICIAP 2022**. *Lecture Notes in Computer Science*, eds S. Sclaroff, C. Distante, M. Leo, G. M. Farinella, and F. Tombari (Cham: Springer), 13233.

[B4] Dadi H. S. Pillutla G. K. M. (2016). Improved face recognition rate using HOG features and SVM classifier. IOSR J. Electron. Commun. Eng. 11, 34–44. 10.9790/2834-1104013444

[B5] Dolhansky B. Bitton J. Pflaum B. Lu J. Howes R. Wang M. Ferrer C. C. (2020). The DeepFake Detection Challenge (DFDC) Dataset. arXiv [preprint] arXiv:2006.07397. 10.48550/arXiv.2006.07397

[B6] Doss C. Mondschein J. Shu D. Fathima G. Sankar S. Davis G.. (2023). Deepfakes and scientific knowledge dissemination. Sci. Rep. 13:13429. 10.1038/s41598-023-39944-3 37596384 PMC10439167

[B7] Ewees A. A. Gaheen M. A. Alshahrani M. Anter A. Ismail F. (2024). Improved machine learning technique for feature reduction and its application in spam email detection. J. Intell. Inf. Syst. 62, 1749–1771. 10.1007/s10844-024-00870-z

[B8] Gangwar A. Dhaka V. S. Rani G. Khandelwal S. Zumpano E. Vocaturo E. (2024). Time and space efficient multi-model convolution vision transformer for tomaton disease detection from leaf images with varied backgrounds. Comp. Mater. Continua 79, 1546–2218. 10.32604/cmc.2024.048119

[B9] Ghorbanpour F. Ramezani M. Fazli M. A. Rabiee H. (2023). FNR: a similarity and transformer-based approach to detect multi-modal fake news in social media. Social Network Analysis Min. 13:56. 10.1007/s13278-023-01065-0

[B10] Heo Y. J. Yeo W. H. Kim B. G. (2023). DeepFake Detection Algorithm Based on Improved Vision Transformer. Appl Intell 53, 7512–7527 10.1007/s10489-022-03867-9

[B11] King D. E. (2009). Dlib-ml: a machine learning toolkit. J. Mach. Learn. Res. 10, 1755–1758. Available online at: https://www.jmlr.org/papers/volume10/king09a/king09a.pdf

[B12] Li Y. Yang X. Sun P. Qi H. Lyu S. (2020). “Celeb-DF: A Large-Scale Challenging Dataset for DeepFake Forensics,” in 2020 IEEE/CVF Conference on Computer Vision and Pattern Recognition (CVPR) (Seattle, WA: IEEE), 3204–3213.

[B13] Lin H. Huang W. Luo W. Lu W. (2023). DeepFake detection with multi-scale convolution and vision transformer. Digital Signal Proc. 134:103895. 10.1016/j.dsp.2022.103895

[B14] Passos L. A. Jodas D. Costa K. A. P. Souza Júnior L. A. Rodrigues D. Del Ser J.. (2024). A review of deep learning-based approaches for deepfake content detection. Expert Systems 41:e13570. 10.1111/exsy.13570

[B15] Rainio O. Teuho J. Klén R. (2024). Evaluation metrics and statistical tests for machine learning. Sci. Rep. 14:6086. 10.1038/s41598-024-56706-x 38480847 PMC10937649

[B16] Ramadhani K. N. Munir R. Utama N. P. (2024). Improving video vision transformer for deepfake video detection using facial landmark, depthwise separable convolution and self attention. IEEE Access 12:8932–8939, 10.1109/ACCESS.2024.3352890

[B17] Rössler A. Cozzolino D. Verdoliva L. Riess C. Thies J. Niessner M. (2019). “FaceForensics++: learning to detect manipulated facial images,” in 2019 IEEE/CVF International Conference on Computer Vision (ICCV) (Seoul: IEEE), 1–11.

[B18] Sagonas C. Antonakos E. Tzimiropoulos G. Zafeiriou S. Pantic M. (2016). 300 faces in-the-wild challenge: database and results. Image Vision Comp. 47, 3–18. 10.1016/j.imavis.2016.01.002

[B19] Tyagi S. Yadav D. (2023). A detailed analysis of image and video forgery detection techniques. Vis. Comput. 39, 813–833. 10.1007/s00371-021-02347-4

[B20] Usmani S. Kumar S. Sadhya D. (2024). Efficient deepfake detection using shallow vision transformer. Multimed Tools Appl. 83, 12339–12362. 10.1007/s11042-023-15910-z

[B21] Wang S. Li B. Khabsa M. Fang H. Ma H. (2020). Linformer: Self-attention with linear complexity. arXiv [preprint] arXiv:2006.04768. 10.48550/arXiv.2006.04768

[B22] Westerlund M. (2019). The emergence of deepfake technology: a review. Technol. Innovat. Managem. Rev. 9, 39–52. 10.22215/timreview/1282

[B23] Yang X. Li Y. Lyu S. (2019). “Exposing deep fakes using inconsistent head poses,” in 2019 IEEE International Conference on Acoustics, Speech and Signal Processing (ICASSP) (Brighton: IEEE), 8261–8265.

[B24] Zandt F. (2024). How Dangerous are Deepfakes and Other AI-Powered Fraud? Statista Daily Data. Available online at: https://www.statista.com/chart/31901/countries-per-region-with-biggest-increases-in-deepfake-specific-fraud-cases/ (accessed March 20, 2025).

